# The Book World of Medicine and Science

**Published:** 1899-04-08

**Authors:** 


### The Book World of Medicine and Science.

Mediterranean Winter Resorts. A Practical Handbook to the Principal Health and Pleasure Resorts on the Shores of the Mediterranean. By Eustace A. Reynolds-Ball, F.R.G.S. (With a Map and several Diagrams. Fourth Edition. London: Kegan Paul, Trench, and Co. 1899. Price 6s.)

This is a very good little book. It tells a great deal, and mostly of the sort that travellers want. Moreover it is easy to read, and not over-crowded with those local details which are more appropriately sought for in the separate local guides issued at every watering-place. Plenty is told, however, about each of the places mentioned to enable the intending visitor to form a very good estimate of the comparative advantages, and even disadvantages, of the various resorts, and sufficient is said about hotels, pensions, &c., to enable the neophyte in the art of wintering abroad to find his way about on arrival in the south. Although bound in one handy volume, and paged consecutively, the book makes a pretence of being in two volumes, which we think is a mistake. Two volumes, two sets of routes, and two indices for one book on what is practically one subject is a somewhat cumbrous arrangement. The first volume treats of the northern shores—the French Riviera, the Italian Riviera and Florence, South Italy, and the south of Spain; while the second volume deals with Morocco, Algeria, Egypt, and the Mediterranean Islands. The general plan adopted in arranging the articles on the various stations seems to have been to deal with the salient points which go to determine the attractiveness or the opposite of any health resort in a definite order. Thus we have climatic conditions, society, hotel and villa accommodation, amusements, sport, "principal attractions," places of interest, and excursions, described in much the same order in regard to each town, making it very easy to find the particular sort of information required. A careful perusal of many of the pages in question shows that the information given is not only very full, but is reliable and up to date. The chapters dealing with the African resorts are especially interesting in view of the comparatively recent development of some of them. Altogether, we consider that to the intending traveller, especially if an invalid, the handbook will be found of great service. The chapter on the medical aspects of the various places, contributed by medical men practising there, are in many cases full and of much interest.

The Diseases of Children. By James Frederic Goodhart, M.D., F.R.C.P. With the assistance of George Frederic Still, M.A., M.D., M.R.C.P. Sixth edition. (London: J. and A. Churchill. 1899. Price 10s. 6d.)

A sixth edition ought not to require much comment, for so continued a demand clearly indicates that the book has supplied a want. A careful perusal of the work as it now appears after its revisal gives the clue to its established popularity. For one thing, it is easy to read, and in these days of dull compilations that is a mercy of no small magnitude; but more especially, as it seems to us, its acceptability is due to its being the outcome of a large personal experience. Perhaps the "I" is a little prominent, and if the book before us were a mere recitation of accepted views it might be out of place, but that is not the case. The views of others are given, but the reader is left in no doubt as to what Dr. Goodhart himself thinks, or as to what he would himself do in given cases, and this imparts a life and interest to the pages which a less personal treatment of the subject would hardly possess. Beginning with a chapter descriptive of the points in which the examination and treatment of children present special peculiarities, chapters follow on growth and dentition, on the diet of children in health, and on diseases caused by diet; then the various diseases to which children are liable are successively dealt with; and finally, as appendices, there are formulæ, recipes, and a copy of the directions given to mothers at the Hospital for Sick Children in regard to the care of children suffering from infantile paralysis. In the chapter on diphtheria we notice that it is said that "so far as is known, there is practically no evidence to show that diphtheria is carried by an intermediary, not actually diseased, to a third person, and therefore, strictly speaking, such children might safely mix with others." Very wisely, however, in our opinion, it is added, "But the interests at stake being great, it is generally advisable that, so long as there is diphtheria in a house, all the children should be regarded as in quarantine." Great stress is laid on the local treatment of diphtheria, but the advantages of antitoxin are also insisted on, and it is pointed out that at the Great Ormond Street hospital it has been found that since the introduction of antitoxin many cases of laryngeal diphtheria with urgent dyspnœa could be tided over this difficulty by intubation where in former days, without antitoxin, the continued spread of membrane would have necessitated tracheotomy. Tracheotomy, by the bye, is a proceeding for which Dr. Goodhart seems to have only a very qualified admiration.

We notice that the very uncouth name Rötheln is used for German measles instead of the official "rubella," and that the term epidemic roseola is given as a synonym for it, while roseola or rose rash is described as something quite different, having no strict right to be considered in association with the specific exanthemata, except, indeed, for the difficulties which arise in diagnosis. It is, perhaps, a pity that the word scarlatina is retained for scarlet fever, especially as it is not done for the sake of uniformity with official nomenclature, the authors showing their independence of official tradition, not only by their use of the term Rötheln, but by speaking of typhoid rather than enteric fever.

The book shows evidence in many places of very careful revision, although here and there a slip may be found, showing that it was originally written some time ago. For example, where we are told, under the head of operation for intussusception, that great care should be exercised not to expose the surface of the child to cold, it is added: "There is a tendency to neglect this precaution in the present age of sprays," &c. Still, take it all round, it is a very interesting and living book and one well worth the reading.

